# Systemic cancer therapy: achievements and challenges that lie ahead

**DOI:** 10.3389/fphar.2013.00057

**Published:** 2013-05-07

**Authors:** Michael O. Palumbo, Petr Kavan, Wilson H. Miller, Lawrence Panasci, Sarit Assouline, Nathalie Johnson, Victor Cohen, Francois Patenaude, Michael Pollak, R. Thomas Jagoe, Gerald Batist

**Affiliations:** Department of Medicine and Oncology, Sir Mortimer B. Davis Jewish General Hospital, Segal Cancer Centre, McGill UniversityMontreal, QC, Canada

**Keywords:** chemotherapy, targeted therapy, hormonal therapy, immunotherapy, toxicity, systemic therapy

## Abstract

In the last half of the century, advances in the systemic therapy of cancer, including chemotherapy, hormonal therapy, targeted therapy, and immunotherapy have been responsible for improvements in cancer related mortality in developed countries even as the population continues to age. Although such advancements have yet to benefit all cancer types, systemic therapies have led to an improvement in overall survival in both the adjuvant and metastatic setting for many cancers. With the pressure to make therapies available as soon as possible, the side-effects of systemic therapies, in particular long-term side-effects are not very well characterized and understood. Increasingly, a number of cancer types are requiring long-term and even lifelong systemic therapy. This is true for both younger and older patients with cancer and has important implications for each subset. Younger patients have an overall greater expected life-span, and as a result may suffer a greater variety of treatment related complications in the long-term, whereas older patients may develop earlier side-effects as a result of their frailty. Because the incidence of cancer in the world will increase over the next several decades and there will be more people living with cancer, it is important to have an understanding of the potential side-effects of new systemic therapies. As an introductory article, in this review series, we begin by describing some of the major advances made in systemic cancer therapy along with some of their known side-effects and we also make an attempt to describe the future of systemic cancer therapy.

## INTRODUCTION

“Vous n’êtes rien qu’un empoisonneur!”…“You are nothing but someone who poisons people!” were the first words a medical oncologist, who was just beginning his practice, heard from a very well respected surgical oncologist at Sir Mortimer B. Davis Jewish General Hospital approximately 30 years ago. Of course, such statements were not rare then, at a time when medical oncology was just beginning to be recognized as a medical specialty. How was anyone to know that medical oncologists, with all their “toxic poisons” would actually have some success stories to describe 30 years down the road? Without a doubt, there have been a number of advances in systemic therapy for a variety of cancers, both in the adjuvant and metastatic setting, which have led to an increase in cure rates and overall survival. Such systemic treatments, to contrast with local therapies such as surgery or radiotherapy, usually fall into the categories of (a) conventional cytotoxic chemotherapy, (b) hormonal agents or (c) targeted therapy or immunotherapy. Although one cannot deny these systemic therapies have been at least partially responsible for improved outcomes, it is also true that their impact has been greatest in a subset of cancers, and that these same treatments are associated with a number of important longer term side-effects. In the first part of this review we summarize the advances achieved in the overall treatment of selected cancers, and detail some of the known long-term side-effects related to conventional chemotherapy and more briefly on those related to hormonal therapy. In the second section of this review, we discuss the rapidly expanding field of targeted and immunotherapy for cancer, and discuss the likely impacts that these new therapeutic agents may have on the profile of cancer treatment in general, and treatment-related toxicities in particular. While it is important to acknowledge that many of the successes in improving cancer care and overall survival result from rapid developments in the use of multimodal treatment regimens, a detailed discussion of the advances in surgical techniques and radiation therapy is beyond the scope of this article.

## CONVENTIONAL CHEMOTHERAPY/CYTOTOXIC AGENTS

With regards to systemic anti-cancer therapy, conventional chemotherapy agents or cytotoxic agents were the first agents in the armamentarium for the war on cancer. Ironically, the real thrust to study these agents occurred after agents such as nitrogen mustard gas were used in World War I and autopsies on soldiers revealed lymphoid hypoplasia and myelosuppression (Gilman and Philips, 1946; Chabner and Roberts , 2005). Over the next several decades numerous cytotoxic agents were discovered. Categories today include, but are not limited to, alkylating agents (e.g., cyclophosphamide, temozolomide, cisplatin, oxaliplatin), anti-metabolites (e.g., methotrexate, cytarabine, fluorouracil, capecitabine), anti-tumor antibiotics (e.g., doxorubicin, epirubicin, bleomycin), topoisomerase inhibitors (e.g., etoposide, irinotecan) and microtubule stabilizers (e.g., paclitaxel, docetaxel). Typically, these agents are not tumor specific and frequently their administration results in significant toxic effects to non-cancerous tissues. The success of these agents relies largely upon their differential toxicity for tumor cells which typically have a high mitotic rate and increased dependence on continuous supply of biomolecules for growth, compared to normal non-cancerous tissues. Over the last several decades, administration of these agents was generally geared at eliminating all cancer cells in the body, often at the expense of significant acute but reversible toxicity in normal tissues. The ability of normal tissues to repair chemotherapy-related damage and recover is key to enabling the patient to tolerate treatment and survive. As demonstrated below, the use of cytotoxic agents in this type of treatment strategy has been particularly successful in a variety of cancers in pediatric and adult patients.

### CANCER IN PEDIATRIC AND YOUNG ADULT PATIENTS

Hodgkin lymphoma (HL) serves as an excellent model to highlight the early successes of chemotherapy, radiation and combined modality therapy, but is also a sobering reminder of the long-term toxicities induced by these treatments in cancer survivors. While HL was universally fatal 30 years ago, it is now curable in 75–80% of patients with systemic chemotherapy (Viviani et al., 2011) consisting of doxorubicin (Adriamycin), bleomycin, vinblastine, and dacarbazine (ABVD). The current approach and challenge in the management of HL is balancing toxicity with efficacy, recognizing that 50% of patients with relapsed HL may be cured with salvage chemotherapy and autologous stem cell transplant (Schmitz et al., 2002). Although cytotoxic chemotherapy likely does play a role in long-term toxicities, radiation therapy is also recognized as being an important contributor. Long-term toxicities associated with HL therapy include secondary solid tumor malignancies and premature cardiovascular disease which have been reported as the leading causes of death in HL survivors (Hodgson et al., 2007; Castellino et al., 2011). Indeed the absolute risk of secondary cancers including breast, lung, gastrointestinal, and thyroid cancers at 25 years for patients treated with both chemotherapy and radiation is ~22% (van Leeuwen et al., 1995, 2003; Sankila et al., 1996; Aisenberg et al., 1997; Swerdlow et al., 2000; Dores et al., 2002; Ng et al., 2002; Aleman et al., 2003; Hodgson et al., 2007). The risk of breast cancer in women treated before the age of 20 has been reported as high as 40% at 10 years (Aisenberg et al., 1997) and the risk of developing secondary myelodysplasia or acute myelogenous leukemia and anthracycline induced cardiotoxicity is ~2 and 7% respectively (Castellino et al., 2011; van der Pal et al., 2012). As a result trials are ongoing to try and determine the optimal dosing strategies to successfully treat HL and limit these long-term side-effects (Castellino et al., 2011).

Pediatric acute lymphoblastic leukemia (ALL), 40 years ago, was a uniformly fatal disease. Today, 75 to 80% of children are cured using a course of combination chemotherapy including an induction, consolidation and maintenance phase along with intrathecal chemotherapy injections (Pui et al., 2004; Gatta et al., 2005). Induction chemotherapy generally consists of steroids along with vincristine and asparaginase. Consolidation therapy follows induction therapy and can include a combination of cytarabine, methotrexate, anthracyclines, alkylating agents, and etoposide. Maintenance therapy can consist of oral 6-mercaptopurine and weekly methotrexate. As more and more patients survive, however, the long-term toxicities are becoming increasingly apparent. As in HL, radiation therapy is believed to play an important role in the development of long-term toxicities. Some of the long-term toxicities recognized to date include moderate to severe neurological or cognitive impairment in 3–15% of patients, a sixfold increase in risk of stroke, a threefold increase in risk of short stature, infertility (in post-pubescent males) and secondary malignancies including brain tumors and secondary leukemia (Ise et al., 1986; Neglia et al., 1991; Relling et al., 1999; von der Weid and Swiss Pediatric Oncology Group, 2001; Bowers et al., 2006; Chow et al., 2007 ). In addition cytotoxic chemotherapy treatment is a recognized cause of “secondary” leukemias, with the risk reaching 5% for most ALL survivors and a 20% risk in survivors of T cell ALL at 6 years (Pui et al., 1989).

Pediatric medulloblastoma is the most common malignant brain tumor of childhood. Over the last 30 years, a multimodality approach including surgery, chemoradiation, and combination chemotherapy has improved overall survival to approximately 80% at 5 years (Packer et al., 2006). Chemotherapy agents used include vincristine, cisplatin with cyclophosphamide or carmustine. Chemotherapy administration has been found to be particularly useful in certain high risk subsets and has also resulted in the use of less radiation therapy in lower risk groups, reducing radiation induced toxicity (Rutkowski et al., 2005). Long-term side-effect studies are few, but there are reports of neurocognitive impairment, secondary central nervous system tumors, hearing loss, and endocrine abnormalities (Devarahally et al., 2003; Fouladi et al., 2005, 2008; Laughton et al., 2008).

Ewing’s sarcoma and osteosarcoma, tumors diagnosed predominantly in the pediatric and young adult populations, have also seen their prognosis improve significantly with the use of chemotherapy. In both localized Ewing’s sarcoma and osteosarcoma, neoadjuvant followed by local therapy and subsequent adjuvant chemotherapy (when indicated) has improved overall survival from approximately 20% using local control measures alone to approximately 65–70% at 5 years (Nesbit et al., 1990; Anninga et al., 2011). Five year survival rates of 20–40% can also be achieved in patients with isolated lung metastasis with the appropriate use of multimodality therapy including chemotherapy (Bacci et al., 1995, 2008). However many of these patients suffer long-term complications and up to 25% mortality at 25 years (Ginsberg et al., 2010). Causes of death include not only relapse of disease but also secondary neoplasms, cardiomyopathy, and pulmonary complications.

Testicular cancer, in large part thanks to a cisplatin-based chemotherapy regimen, is considered to be the most frequently cured solid tumor. Even patients with advanced disease including brain metastasis have a good chance at cure. Recent reports suggest that over 95% of males diagnosed with the disease are cured after 10 years of follow-up (Siegel et al., 2011). In general, bleomycin, etoposide, and cisplatin (BEP) chemotherapy, remain the standard of care for the disease in particular for patients with advanced disease. However, 20–40% of long-term survivors can suffer some form of otoxicity, neuropathy, and nephropathy (Bokemeyer et al., 1998; Fossa et al., 2002; Brydoy et al., 2009). Bleomycin lung injury can also be quite severe and up to 40% of patients will have some form of toxicity in the first 3 years post-chemotherapy. Most of the cases are mild but up to 10% of cases can develop pulmonary fibrosis and up to 10% of those can eventually die of their lung disease (O’Sullivan et al., 2003).

### CANCER IN ADULT PATIENTS

Tumors such as colon and breast cancer have also seen an improvement in survival resulting from the use of conventional chemotherapy in both the adjuvant and metastatic setting. Stage III colon cancer patients treated with 6 months of 5-fluorouracil (5-FU)-based chemotherapy in combination with oxaliplatin have improved 5-year disease free survival rates by 20–30% (Andre et al., 2004, 2009). In the metastatic setting, with isolated liver or pulmonary metastasis, 5 year overall survival rates in the 20–40% range have been reported with multimodality therapy including chemotherapy (Adam et al., 2009; Masi et al., 2009). Median overall survival in the non-operable metastatic colon cancer has also improved, and now stands at approximately 2 years with the use of 5-FU-based chemotherapy with oxaliplatin and irinotecan. In adjuvant breast cancer, combination chemotherapy frequently containing an anthracycline with a taxane has improved 5 year disease free survival rates by up to 20%(Berry et al., 2006)depending on the initial stage of disease and tumor characteristics. Similarly, a wide number of chemotherapy agents are known to be effective in treating breast cancer and their use, has improved overall survival in the metastatic setting. Unfortunately, despite huge improvements in the control of acute chemotherapy-related symptoms such as chemotherapy-induced nausea and vomiting, other more insidious or persistent symptoms such as fatigue and neuropathy have proven more challenging to address. Thus benefits in overall survival in colon cancer due to use of agents such as oxaliplatin, have to be weighed against the impact of persistent peripheral neuropathy which can affect up to 10% of patients 4 years post-treatment with 1% having severe symptoms (Andre et al., 2009). Similarly adjuvant chemotherapy in breast cancer survivors is associated with fatigue, neuropathy, cardiomyopathy, ovarian dysfunction, leukemia, and psychological distress (Postma et al., 1995; Partridge and Ruddy, 2007; Beadle et al., 2009; Bowles et al., 2012).

### HORMONAL THERAPY

Hormonal therapy has also played an important role in the treatment of breast and prostate cancer. Hormonal therapy in breast cancer has improved disease free survival in ER/PR+ disease by up to 10% at 10 years (Dowsett et al., 2010; Early Breast Cancer Trialists’ Collaborative Group et al., 2011). In the metastatic setting, use of hormonal therapy (when appropriate and often combined with chemotherapy) has likely improved median overall survival to approximately 2 years with reports of long-term survivors of 8 years and longer (Gennari et al., 2005; Chia et al., 2007). Adjuvant hormonal therapy, depending on whether tamoxifen or an aromatase inhibitor (AIs) is used tends to be associated with musculoskeletal discomfort, early osteoporosis and increased cardiac events for AIs versus increased venous thromboembolic events and uterine hyperplasia (rarely malignancy) with tamoxifen (Mincey et al., 2006; Cuppone et al., 2008; Schaapveld et al., 2008; Amir et al., 2011). With regards to prostate cancer, in both the adjuvant setting for locally advanced disease and in overt metastatic disease, androgen deprivation therapy is associated with an improvement in overall survival (Nguyen et al., 2011; Hussain et al., 2012). Side-effects from androgen deprivation therapy include sexual dysfunction, hot flashes, early osteoporosis complicated by bone fractures and loss of lean body mass (Shahinian et al., 2005; Wilke et al., 2006; Smith et al., 2012).

## THE FUTURE OF CONVENTIONAL CHEMOTHERAPY

The examples described above demonstrate that conventional cytotoxic chemotherapy has an important role in the management of patients diagnosed with cancer, but the use of these agents is clearly associated with long-term toxicities in long-term survivors. Moreover we can anticipate that further improvements in cancer survival related to the increasing use of cytotoxic chemotherapies in more potent and effective regimens, will also lead to increasing numbers of patients being affected by the cancer treatment-related long-term toxicities. Present and future challenges include identifying the appropriate dosing of cytotoxic agents (and other components of multimodal anti-cancer treatment regimens) to maximize therapeutic efficacy while limiting both acute and long-term side-effects. Individualized assessments of a patients’ risk *of cancer-related death/recurrence* is another important conceptual approach that is being used more and more to adapt the aggressiveness of the treatment strategy thus attempting to balance the need for cancer treatment and the likelihood of treatment-related long-term toxicities. Indeed a number of trials are ongoing to answer these questions but these are statistically complex trials that require a large number of subjects as well as extended follow up (several years) to accurately capture long-term toxicities. An important group of patients which require special consideration is the rapidly expanding population with metastatic incurable disease, where chemotherapy treatment is frequently used to control cancer indefinitely. The sequential use of several different chemotherapy agents is becoming increasingly common to overcome tumor resistance as it emerges e.g., in patients with metastatic colon and breast cancer, and there is evidence for improved overall survival. Nevertheless these patients often accumulate considerable exposure to multiple chemotherapeutic agents, and are thus at high risk of cumulative treatment-related side-effects. Paradoxically patient ability or willingness to tolerate such side-effects, rather than uncontrolled disease, or absence of potential active anti-cancer treatments, may rapidly become the limiting factor for treatment success in this population.

## TARGETED THERAPY AND IMMUNOTHERAPY

Although the use of cytotoxic chemotherapy has had some notable success as described above, a significant proportion of patients suffering from cancer either do not respond to conventional chemotherapy or relapse after treatment. In addition, several cancer types, such as melanoma, renal cell carcinoma, hepatocellular carcinoma, and gastrointestinal stromal tumors (GIST) have very limited response to conventional cytotoxic chemotherapy. For these cancers, patients have frequently received treatments with minimal benefit but significant toxicity, in the hope that this would slow disease progression in some cases. More recently the ability to characterize specific gene mutations in different cancers, and a greater biological understanding of the cellular events and pathways driving carcinogenesis, has led to the development of targeted agents and immunotherapeutics. These new therapies appear to offer considerable advantages in delivering growth inhibitory or cytotoxic effects in a much more cell-specific manner. Furthermore these drugs do not induce the same profile of acute toxicities such as myelosuppression and nausea and vomiting which accompany many of the conventional non-targeted cytotoxic drugs. As described below some of the newer targeted agents have proven highly effective anti-cancer treatments, and have already delivered startling improvements in survival for certain cancers. However, many of the cellular pathways which are disrupted in cancer cells and targeted by these newer treatments, are also of fundamental importance to normal growth or homeostasis in all cells. Furthermore typical treatments are prolonged, extending over many months or years. Both these factors suggest there is a reasonable chance that the targeted agents still carry a risk of effects in other non-cancer cells. In many cases there is still not enough clinical experience with these drugs to establish precise or robust long-term toxicity profiles, but as detailed below, many of these agents do have unforeseen shorter term side-effects and induce variable degrees of morbidity as a result.

### IMATINIB AND THE TREATMENT OF CHRONIC MYELOGENOUS LEUKEMIA

The impact of Imatinib on outcomes in chronic myelogenous leukemia (CML) is perhaps the best example of the marked improvements that targeted treatments have made in cancer treatment. A gene translocation event involving the gene for BCR-ABL creating a constitutively active tyrosine kinase is a central event in the genesis of CML. Imatinib, one of a family of tyrosine kinase inhibitor (TKI) molecules has proven highly effective in the treatment of CML and has had a dramatic impact on the course of this disease. Prior to imatinib, the majority of patients diagnosed with CML in the chronic phase would generally progress to a blastic phase within 5 years of diagnosis and most would die. Since the introduction of imatinib, the 8 year follow-up of the IRIS study has described progression free and overall survival of greater than 80% in patients treated with imatinib during chronic phase CML (Deininger et al., 2009). It is now estimated that the majority of patients with CML have a normal life expectancy (Gambacorti-Passerini et al., 2011). For the most part, however, these patients are not considered cured, rather imatinib maintains a very low level of CML cells and lifelong therapy may be required. Many consider imatinib to be the most successful of all the targeted therapies discovered to date against cancer. This success largely relates to the relatively simple molecular biology of CML, which unlike most other cancers, is associated with one dominant single gene rearrangement resulting in its neoplastic potential.

Imatinib has also been found to be quite active in treating GIST both in the adjuvant and locally advanced as well as metastatic setting (Blanke et al., 2008; Joensuu et al., 2012). GIST frequently overexpresses c-kit and imatinib cross-reacts and significantly inhibits this tyrosine kinase receptor, and as a result relapse-free survival and progression-free survival have significantly improved in this previously chemotherapy-refractory disease. In advanced disease or metastatic disease, complete responses are rare but long-term stable disease on imatinib, can be maintained for 2–3 years. In the adjuvant setting, it is presently not clear how long patients should remain on imatinib post-resection and some data suggests the longer the better (Joensuu et al., 2012).

Interestingly, even with the relative selectivity of imatinib, patients still suffer from a number of side-effects that are likely from cross-reactivity of the agent with other tyrosine kinases on healthy tissues. The side-effects of imatinib are generally considered mild but may persist for as long as the patient remains on therapy and have been associated with a diminished quality of life (Efficace et al., 2011). Side-effects include but are not limited to fatigue, diarrhea, nausea and vomiting along with muscle cramps, musculoskeletal pains, rash and edema. Other toxicities include cytopenias, hepatotoxicity, and cardiac toxicity. Long-term toxicities of imatinib and the long-term consequences of the chronic side-effects described above are not yet known but it should be noted that after 8 years of follow-up in patients being treated for CML, no increased risk of secondary cancers has been described (Verma et al., 2011).

### THE ROLE OF TKIs IN NON-SMALL CELL LUNG CANCER

In the last decade, a number of other TKIs have been developed and have shown some success in treating lung cancers. Erlotinib and gefitinib are TKIs that target a mutated active epidermal growth factor receptor that is implicated in approximately. 10-15% of advanced non-small cell lung cancer (NSCLC) tumors. First-line treatment with either gefitinib or erlotinib, in patients harboring an EGFR mutation with stage IV disease is associated with an approximately 5–6 month improvement in progression-free survival when compared to conventional chemotherapy (Mok et al., 2009; Fukuoka et al., 2011; Zhou et al., 2011; Rosell et al., 2012). Benefit in overall survival has been difficult to demonstrate given many trials allowed cross-over and the majority of patients went on to get 2nd line therapy. Toxicities reported to date include fatigue, diarrhea, and rash along with hepatic toxicity and rare events of hepatic failure and interstitial pneumonitis. (Liu et al., 2007; Pellegrinotti et al., 2009). Crizotinib, is another TKI approved in NSCLC after studies have shown marked and durable responses in patients harboring an anaplastic lymphoma kinase (ALK) fusion oncogene (Kwak et al., 2010). The ALK fusion oncogene is thought to be present in approximately 2–7% of patients with NSCLC but as is the case for EGFR mutations,. ALK is predominantly present in young non-smoking patients. Similar to other TKIs, side-effects associated with crizotinib include fatigue, diarrhea, nausea, and vomiting which are rarely severe but there are reports of life-threatening hepatotoxicity and interstitial pneumonitis (Ou et al., 2011; Weickhardt et al., 2012). As with gefitinib and erlotinib, data regarding crizotinib associated long-term side-effects is very limited given the short period of time they have actually been approved for treatment.

### TARGETED ANTI-CANCER TREATMENTS FOR RENAL CELL CARCINOMA

With limited efficacy of conventional chemotherapy, targeted therapy has been the predominant focus in renal cell cancers (RCCs) in particular for clear cell renal cancers. Two classes of agents, TKIs (predominantly inhibiting vascular endothelial growth factor tyrosine kinases, vascular endothelial growth factor receptor (VEGFR), but most are multi-targeted TKI) and mammalian target of rapamycin (mTOR) inhibitors are the key players. VEGFR-associated pathways have also been shown to have an important role in clear cell RCC and other cancers, and VEGFR inhibitors have essentially become the 1st line therapy in metastatic RCC for most patients. Sunitinib, pazopanib, axitinib, and sorafenib are VEGFR inhibitors that have demonstrated therapeutic efficacy in metastatic clear cell RCC (Escudier et al., 2009; Motzer et al., 2009; Sternberg et al., 2010; Rini et al., 2011). The mTOR inhibitors, temsirolimus, and everolimus, have also shown activity in metastatic clear cell RCC (Hudes et al., 2007; Motzer et al., 2008, 2010). Most patients with metastatic RCC will be started on one of these classes of agents often resulting in either stability of disease or some degree of partial response. In almost all cases resistance eventually develops, generally within 8 months, and 2nd line therapy with a different agent either in the same class or different class (i.e., VEGFR inhibitors or mTOR inhibitors) is started. The majority of patients inevitably remain on such therapies lifelong. The VEGFR inhibitors tend to have a number of associated toxicities but to date those reported are mostly for sunitinib and sorafenib simply because the other agents have less available data. Side-effects include hypertension, an increase in arterial thromboembolic events, thyroid dysfunction, cutaneous toxicity, cardiotoxicity, hepatotoxicity, and skeletal muscle wasting (Motzer et al., 2007; Rini et al., 2007; Lacouture et al., 2008; Zhu et al., 2009; Choueiri et al., 2010). Although controversial, pre-clinical studies in mice have raised concerns that, sunitinib, a multi-targeted TKI which inhibits VEGF signalling may lead to paradoxical enhancement of tumor metastatic growth in some tumor models (Iacovelli et al., 2012). It should be stressed, however, that this potential drawback of sunitinib has not been demonstrated in humans. Temsirolimus is associated with rash, nausea, vomiting, and anorexia in 30–50% of patients but severity is considered mild in most patients (Hudes et al., 2007). Everolimus can result in severe lymphopenia, hyperglycemia, and stomatitis (Motzer et al., 2008, 2010). Interstitial pneumonitis is reported to occur anywhere between 0.5 and 10% of patients with both everolimus and temsirolimus (Iacovelli et al., 2012). For both classes, a description of long-term side-effects is limited by their short duration of time in use.

### THE USE OF MONOCLONAL ANTIBODIES FOR ANTI-CANCER TREATMENTS

Targeted therapy agents also include a number of monoclonal antibodies. Examples include trastuzumab, bevacizumab, panitumumab, and cetuximab. In the adjuvant setting trastuzumab, a monoclonal antibody against Her2 (an EGFR receptor overexpressed in approximately 20% of breast cancers), has improved the overall survival in women with breast cancers overexpressing Her2, by 5–10% at 5 years (Slamon et al., 2011). It has also been shown to improve overall survival in women suffering from metastatic disease (Slamon et al., 2001; Marty et al., 2005). In terms of side-effects, heart failure appears to be the only major concern and occurs in less than 5% of patients but generally is reversible if caught early and treatment discontinued (Perez et al., 2008).

As mentioned previously, the VEGFR pathway is believed to play an important role in a variety of cancers. Bevacizumab is a monoclonal antibody against the ligand of VEGFR, and its use has been shown to increase overall survival by approximately 4 months in metastatic colon cancer when used in combination with irinotecan (Hurwitz et al., 2004). However, a notable increase in grade three and four adverse reactions associated with bevacizumab were also seen which include hypertension, bowel perforation, arterial thromboembolic events, impaired wound healing, and bleeding events (Kozloff et al., 2009). Addition of bevacizumab to 5-FU-based chemotherapy in the adjuvant setting has not shown any benefit.

Panitumumab and cetuximab are anti-EGFR antibodies that have been shown to increase overall survival in metastatic colon cancer by approximately 5 months in chemotherapy refractory metastatic colon cancer (Van Cutsem et al., 2007, 2008; Karapetis et al., 2008). These antibodies target the EGFR receptor, K-ras, and benefit patients that have a wild-type K-ras gene which is found in approximately 40% of patients with colon cancer. Adverse reactions from these antibodies include injection reactions, rash, diarrhea, venous thromboembolic events, and electrolyte abnormalities. Similarly to bevacizumab, addition of either antibody in addition to standard combination chemotherapy has not shown any benefit in the adjuvant setting.

### TARGETED ANTI-CANCER TREATMENTS AND IMMUNOTHERAPY FOR MALIGNANT MELANOMA

For the last 30 years or more attempts to use conventional cytotoxic chemotherapy in the treatment of malignant melanoma resulted in minimal benefit. Two recent major advances in melanoma include the development of a family of TKIs termed Braf inhibitors, and a breakthrough immunotherapeutic agent referred to as ipilimumab. Both have demonstrated significant benefit in overall survival in patients with metastatic melanoma (Hodi et al., 2010; Chapman et al., 2011; Robert et al., 2011). Studies in the adjuvant setting, however, are ongoing. Braf inhibitors target mutated Braf tyrosine kinase receptors. Approximately 50% of patients with cutaneous melanoma have activating Braf mutations. Use of Braf inhibitors in patient’s harboring the mutated oncogene results in dramatic disease regressions in >95% of patients. Unfortunately, resistance eventually develops in between 6 and 12 months for the majority of patients. Ipilimumab is an anti-CTLA-4 antibody that results in the stimulation of an immune response against melanoma. Approximately 20–30% of patients have some degree of response with approximately 10% having durable remissions for several years. Side-effects of Braf inhibitors include arthralgia, fatigue, rash and an increase in the number of squamous cell carcinomas and keratoacanthomas. Side-effects from ipilimumab are predominantly related to an inflammatory type autoimmune response on normal body tissues and include enterocolitis, hepatitis, dermatitis, and endocrinopathies.

## THE FUTURE OF TARGETED AND IMMUNOTHERAPY

Targeted therapy is heralded by many as the holy grail of cancer treatment, the best example of which is imatinib in the treatment of CML. In some instances, however, targeted therapies have fallen short of expectations. Despite showing an improvement in progression free and overall survival, resistance to these agents can develop sometimes within a short time of initiation of therapy. The limited efficacy of these agents in some circumstances likely reflects less on the lack of sophistication of the targeted agents and more on the genetic instability of tumors and the presence of tumor subclones which develop resistance or are inherently resistant to these therapies. Further genetic characterization of tumors may help to ensure that the right therapy is used in the right patient. Additionally, a number of these targeted therapies have important side-effect profiles, some being associated with pulmonary fibrosis, gastrointestinal toxicity, hepatotoxicity, cardiomyopathy, and other cardiovascular complications. Whilst these may well be tolerable or acceptable in the short term, where patients are required to remain on such therapies lifelong the impact on function and quality of life may be more difficult to sustain. Despite the many promising and exciting advances in this area of cancer therapeutics there are also many areas of uncertainty including whether the efficacy demonstrated by imatinib in CML can ever be replicated with other agents in other cancer types; and what the long-term health consequences of remaining on these types of therapies will be. Meanwhile, though optimum treatment regimens and dosing schedules using targeted therapies are still to be established, lifelong treatment for many patients seems to be a likely outcome. Hence the development of new and highly effective targeted anti-cancer therapies, also brings with it new challenges: namely how to mitigate not only short-term transient toxicities, but also the persistent or late side-effects which emerge when patients have to remain on these therapies for extended periods. Addressing these challenges will be vital to the continued success of this collective war on cancer.

## Conflict of Interest Statement

The authors declare that the research was conducted in the absence of any commercial or financial relationships that could be construed as a potential conflict of interest.
